# Neurometabolite mapping highlights elevated myo-inositol profiles within the developing brain in down syndrome

**DOI:** 10.1016/j.nbd.2021.105316

**Published:** 2021-06

**Authors:** Prachi A. Patkee, Ana A. Baburamani, Katherine R. Long, Ralica Dimitrova, Judit Ciarrusta, Joanna Allsop, Emer Hughes, Johanna Kangas, Grainne M. McAlonan, Mary A. Rutherford, Enrico De Vita

**Affiliations:** a Centre for the Developing Brain, School of Biomedical Engineering and Imaging Sciences, King's College London, St. Thomas' Hospital, London SE1 7EH, UK; b Centre for Developmental Neurobiology, Institute of Psychiatry, Psychology and Neuroscience, King's College London, SE1 1UL, UK; c MRC Centre for Neurodevelopmental Disorders, King's College London, SE1 1UL, UK; d Department of Forensic and Neurodevelopmental Science, Sackler Institute for Translational Neurodevelopment, Institute of Psychiatry, Psychology and Neuroscience, King's College London, SE5 8AB, UK; e Department of Biomedical Engineering, School of Biomedical Engineering and Imaging Sciences, King's College London, St. Thomas' Hospital, London SE1 7EH, UK

**Keywords:** Developing, Brain, Down syndrome, MR spectroscopy, Mass spectrometry, Myo-inositol

## Abstract

The neurodevelopmental phenotype in Down Syndrome (DS), or Trisomy 21, is variable including a wide spectrum of cognitive impairment and a high risk of early-onset Alzheimer's disease (AD). A key metabolite of interest within the brain in DS is Myo-inositol (mIns). The NA+/mIns co-transporter is located on human chromosome 21 and is overexpressed in DS. In adults with DS, elevated brain mIns was previously associated with cognitive impairment and proposed as a risk marker for progression to AD. However, it is unknown if brain mIns is increased earlier in development.

The aim of this study was to estimate mIns concentration levels and key brain metabolites [*N*-acetylaspartate (NAA), Choline (Cho) and Creatine (Cr)] in the developing brain in DS and aged-matched controls. We used *in vivo* magnetic resonance spectroscopy (MRS) in neonates with DS (*n* = 12) and age-matched controls (*n* = 26) scanned just after birth (36–45 weeks postmenstrual age). Moreover, we used Mass Spectrometry in early (10–20 weeks post conception) *ex vivo* fetal brain tissue samples from DS (*n* = 14) and control (*n* = 30) cases.

Relative to [Cho] and [Cr], we report elevated ratios of [mIns] *in vivo* in the basal ganglia/thalamus, in neonates with DS, when compared to age-matched typically developing controls. Glycine concentration ratios [Gly]/[Cr] and [Cho]/[Cr] also appear elevated. We observed elevated [mIns] in the *ex vivo* fetal cortical brain tissue in DS compared with controls.

In conclusion, a higher level of brain mIns was evident as early as 10 weeks post conception and was measurable *in vivo* from 36 weeks post-menstrual age. Future work will determine if this early difference in metabolites is linked to cognitive outcomes in childhood or has utility as a potential treatment biomarker for early intervention.

## Highlights

• Myo-inositol is elevated in fetal cortical DS brain.
• Elevated myo-inositol was detected in the neonatal DS brain using MR spectroscopy.
• Elevated Choline/Creatine and Glycine/Creatine was also present in DS neonatal brain.

## 1 Introduction

Down Syndrome (DS) is a complex genetic condition, resulting from the triplication of human chromosome 21 (HSA21; (Trisomy 21), and is associated with a wide spectrum of neurodevelopmental outcomes (Karmiloff-Smith et al., 2016; Baburamani et al., 2019). Individuals typically present with cognitive deficits, and variable impairments in speech, language and motor functions, coupled with a high risk of early-onset dementia and Alzheimer's disease (AD) (Rachidi and Lopes, 2007; Wiseman et al., 2015; Karmiloff-Smith et al., 2016). There is limited understanding of brain development in DS, but early alterations may contribute to the variations in lifelong neurodevelopmental outcomes and ultimately to the risk for early-onset AD. Recent *in vivo* studies have demonstrated altered structural brain development in DS from as early as 20 weeks gestation, but it is not known whether these are associated with abnormal metabolic profiles (Patkee et al., 2020). Metabolic derangements, specifically with an elevation of myo-inositol (mIns), have been described across development in mouse models of DS, as well as in the child and adult human brain with DS, but there have been no studies assessing brain metabolism at much younger ages.

MIns is of particular interest as the NA+/mIns co-transporter (SLC5A3) is located on HSA21 and is overexpressed in DS (Berry et al., 1999). MIns is a pentose sugar, ubiquitously distributed in brain tissue and is known to be involved in neuronal signalling and development, osmoregulation, membrane metabolism, and amyloid deposition (Beacher et al., 2005; Zhu and Barker, 2011; Blüml et al., 2013). Magnetic resonance spectroscopy (MRS) studies in the adult DS population have reported significantly elevated ratios of mIns in DS brains, with and without the neuropathological hallmarks of AD, as compared with healthy adult controls (Lin et al., 2016). Higher levels of mIns in adults with DS, and those with both DS and AD, have been found to correlate with poor cognitive performance, suggesting that this metabolite might have utility for monitoring response to treatment and/or constitute a treatment target (Lin et al., 2016). Consistent with the latter, studies in mouse models of DS have shown that higher levels of mIns could be reduced by treatment with lithium, which also rescued both synaptic plasticity and memory deficits (Huang et al., 1999; Contestabile et al., 2013). Whilst such studies have made valuable first steps to revealing a link between the metabolite profile of DS and functional outcomes, it is unknown when this alteration in mIns (and/or other metabolites supporting brain function) emerges.

Current therapeutic approaches to improve cognition in DS, target children and adults; and have so far had limited success. For instance, a clinical trial of lithium supplementation in adults with DS did not report efficacy (Murphy, 2011). Arguably however, intervention may be needed at an earlier timepoint. Identification of neurological differences much earlier in the life course in DS, could potentially offer opportunities for more effective intervention. We and others have recently demonstrated that fetuses and neonates with DS have reduced whole brain, cortical and cerebellar volumes from as early as 20 weeks gestational age (GA), using *in vivo* structural MRI (Tarui et al., 2019; Patkee et al., 2020). Whether such anatomical differences are accompanied by early differences in the metabolite levels, that reflect neuronal and glial function, and alter neurodevelopmental outcome, remains unknown.

The aim of this study was to determine whether the brain metabolite profile and specifically mIns concentration ([mIns]) is already altered in DS during early development *in utero.* We used *in vivo* MRS to assess the metabolic profile of brain tissue (basal ganglia and thalami) in neonates with DS compared to typically developing controls. To complement this, we analysed the metabolite profile in post-mortem fetal cortical brain tissue using Mass Spectrometry. In addition to mIns, other metabolites of interest we focused on were *N*-acetylaspartate, Choline, Creatine and Glycine. We hypothesised that there would be significant alterations in metabolic ratios between the DS and control groups, with increases in mIns levels specifically, in the brain of neonates with DS and secondly, increases in absolute mIns at earlier gestations in brain tissue from fetuses with DS compared with controls.

## 2 Methods

### 2.1 Magnetic resonance spectroscopy study

Ethical approval for this study was obtained from the West London and GTAC Research Ethics Committee (REC) for DS participants (07/H0707/105); and from the Dulwich NREC for the control population (12/LO/2017). Informed consent was obtained from the legal guardians of all infants, prior to imaging at St. Thomas' Hospital, London, UK. All methods were carried out in accordance with the relevant guidelines and regulations.

### 2.2 Participants

Participants who had previously had a fetal scan (Patkee et al., 2020) and consented during their initial scan to be contacted post-delivery, were invited for a neonatal scan up to 46 weeks post-menstrual age (PMA). Neonates with confirmed DS, following genetic karyotyping, were also recruited from the neonatal unit or postnatal wards at St Thomas' Hospital. Participants with DS, some of whom had other non-brain congenital abnormalities such as cardiac defects or gastrointestinal malformations, were included in this study. Clinical details for the neonates with DS can be found in Table 1. Control group neonates were recruited from South London and South East of England antenatal centres as part of the Brain Imaging in Babies Study (BIBS) and scanned at the Centre for the Developing Brain (St. Thomas' Hospital, London, UK). Neonates were included as controls if they had a normal brain appearance on the neonatal MRI, with no other congenital or chromosomal abnormalities, and an uneventful delivery, as acute birth-related events could potentially change metabolite composition even in the presence of normal MR imaging (Lally et al., 2019). Additionally, all control neonates had no known immediate family members with any neurodevelopmental or behavioural conditions (*e.g.* autism and ADHD).

**Table 1:** Age at scan and clinical characteristics of neonates with DS. Gestational age (GA), post-menstrual age (PMA), atrioventricular septal defect (AVSD), atrial septal defect (ASD), ventricular septal defect (VSD) and patent ductus arteriosus (PDA).

Clinical details of the DS cohort					
DS Subject	Age at birth (GA weeks)	Neonatal scan (PMA weeks)	Sex (Male/Female)	Cardiac abnormality	Other medical conditions
1	38.86	45.57	F	Tetralogy of Fallot	Hirschsprung's disease
2	36.43	38.43	F	Tetralogy of Fallot, PDA	x
3	37.71	43.57	F	AVSD, small left ventricle, hypoplastic arch	x
4	38.43	39.71	M	AVSD and hypoplastic aortic arch	x
5	36.71	44.57	M	x	x
6	37.00	37.57	M	AVSD and coarctation of the aorta	x
7	35.29	38.86	M	ASD	Hirschsprung's disease
8	37.00	37.86	F	AVSD	x
9	37.14	38.00	M	x	x
10	39.43	39.43	M	AVSD	x
11	32	32.43	F	mildly dysplastic aortic valve	x
12	31.71	34.14	M	x	x

### 2.3 *In vivo* MRI acquisitions

MR scanning of the DS and control neonates was performed on a Philips Achieva 3 Tesla system (Best, The Netherlands) at the Centre for the Developing Brain (St. Thomas' Hospital, London, UK) using a custom built 32-channel neonatal head coil (Hughes et al., 2017). Infants were placed in supine position, secured within the scanning shell and a neonatal vacuum moulded pillow was used to stabilise the head to reduce movement (Hughes et al., 2017). Auditory protection comprised of earplugs moulded from silicone-based putty placed in the outer ear (President Putty, Coltene/Whaledent Inc., OH, USA), neonatal earmuffs over the ear (MiniMuffs, Natus Medical Inc., CA, USA) and an acoustic hood placed over the scanning shell. Sedation was not administered, and all babies were scanned during natural sleep, following a feed. Pulse oximetry, heart rate, and temperature were monitored throughout the scan and an experienced neonatologist or neonatal nurse attended all examinations. The total examination time for each neonate, including clinical scans, volumetric MRI as well as MRS, did not exceed 60 min.

For volumetric/structural imaging T2-weighted images were acquired in the sagittal and axial planes using a multi-slice turbo spin echo sequence. The stacks of 2D slices were acquired with the following parameters: repetition time TR = 12,000 ms; echo time TE = 156 ms; 125 slices, slice gap = −0.8 mm (overlapping slices); flip angle 90°; acquired voxel size: 0.8 × 0.8 × 1.6 mm^3^ (Hughes et al., 2017). T2-weighted images were reviewed to assess image quality and those with excessive motion. A specialist perinatal radiologist evaluated all neonatal MR images to exclude additional anomalies and confirm appropriate appearance for gestation. Image datasets showing an overt additional structural malformation or brain injury were excluded. Findings from volumetric analysis of these data have been previously reported (Patkee et al., 2020).

### 2.4 *In vivo* MRS acquisition

**Fig. 1:**
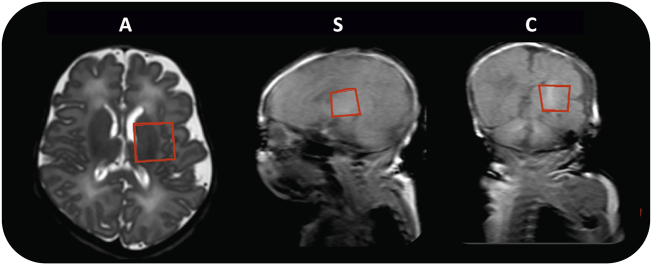
MRS acquisition region of interest. Example of region of interest size 20 × 20 x 20 mm^3^ for MRS, positioned over the left basal ganglia and thalamus. This is shown superimposed on an axial T2-weighted image (A), plus on sagittal (S) and coronal (C) T1-weighted gradient echo pilot scans.

A PRESS sequence was used for MRS. A 20 × 20 × 20 mm^3^ region of interest (ROI) was positioned centrally over the left basal ganglia and thalamus (Fig. 1). This was chosen as substantial literature on early brain development MRS exists for this brain region (Kim et al., 2006; Tomiyasu et al., 2013; Lally et al., 2019).

The PRESS acquisition had an echo time TE = 55 ms (with partial echo times TE1/TE2 = 26/29 ms) and a repetition time TR = 1.5 s; 128 averages were collected over 3 min 18 s and averaged in blocks of 4, resulting in 32 saved spectra. Water suppression was performed with the manufacturer's water-frequency selective excitation scheme (2 radio frequency pulses followed by crusher gradients), which reduced water signal by approximately 99%. No data was acquired without water suppression.

### 2.5 MRS data processing and estimation of metabolite ratios

The manufacturer scanner software performs only a basic automatic processing for immediate quality assessment of the acquired spectra. All spectra were processed offline as described below.

Spectra were imported in Suspect (https://suspect.readthedocs.io/en/latest/)(Rowland et al., 2017) and frequency/phase correction was performed.

Outlier rejection was based on subjective evaluation of the main peaks of Choline, Creatine and *N*-acetylaspartate (respectively at ~3.19, 3.03, 2.01 ppm), and of the residual water signal following spectral registration. Spectra were removed if: (a) the water signal showed abrupt changes or continuous changes beyond 30% of the typical amplitude of initial stable spectra and, (b) the corresponding metabolite peaks had significantly different amplitudes or could not be aligned/phased with the previous ones, potentially indicating subject movement All remaining spectra were averaged.

Metabolite concentration quantification was performed using Tarquin, version 4.3.11 (http://tarquin.sourceforge.net/ (Wilson et al., 2011). With partial echo times TE1 and TE2 specified, Tarquin calculates appropriate PRESS basis set spectra for spectral modelling and fitting. The basis set *1h_brain_gly_cit_glth* provided by Tarquin was used; this includes the following metabolites: Alanine, Aspartate, Citrate, Creatine, GABA, GPC, Glucose, Glutamine (Gln), Gluthatione, Glutamate (Glu), Glycine (Gly), mIns, Lactate, NAA, NAAG (*N*-acetylaspartylglutamate), Phospho-Choline (PCh), Phospho-Creatine (PCr), Scyllo-inositol, Taurine, Glx (Glu + Gln), Creatine CH2, 4 lipid resonances and 4 macromolecular resonances.

In the absence of water reference spectra, absolute metabolite concentrations estimates were not comparable between subjects. We report the following metabolite concentration ratio estimates: [mIns]/[Cho], [mIns]/[Cr], [Cho]/[Cr], [NAA]/[Cr] [Gly]/[Cr], (where for [Cho], [Cr], [NAA] we intend in the following the tCho, tCR, tNAA reported by Tarquin *i.e.*, tCho = PCh + GPC, tCr = Cr + PCr, and tNAA = NAA + NAAG respectively).

As spectral quality assurance criteria, we report the signal to noise ratio (SNR) and the full width at half maximum (FWHM) reported by Tarquin, together with the % standard deviation of the metabolite concentration estimates (%SD) calculated as Cramer Rao lower bounds (Wilson et al., 2011). We also performed a qualitative assessment of the overall fit.

### 2.6 Mass spectrometry

#### 2.6.1 Fetal brain tissue samples

Human fetal cortex material was provided by the Joint MRC/Wellcome Trust (MR/R006237/1) Human Developmental Biology Resource (HDBR) (www.hdbr.org). HDBR provided fresh fetal cortex tissue that was snap-frozen for DS (*n* = 14) and age-matched controls (*n* = 30) in the fetal age range 10 to 20 post-conception weeks (PCW). Genetic diagnosis confirmed presence of Trisomy 21 (DS). There were no other known abnormalities in either group. HDBR specific ethics approval (18/LO/0822, 18/NE/0290) was sought, and tissue was collected following termination of pregnancy, following Human Tissue Act (HTA) regulations. Cortical tissue was then selected, snap frozen and stored at −80 °C. Cases that were used are detailed in Supplementary Table 1.

#### 2.6.2 Sample preparation

Human fetal cortex tissue samples were stored at −80 °C. The cortical tissue used included a variety of regions, as due to the nature of these fetal samples it is often difficult to identify the exact cortical region tissue originates from across the gestational ages. To ensure this did not affect the results, cortical tissue was randomly assigned for the mass spectrometry analysis in both DS and control groups. Approximately 0.1 g of brain tissue from the cortex was collected on dry ice and weighed. 1 ml of HPLC grade Methanol (Sigma, UK) was added and samples were then homogenised for 20 min at 30 Hz using TissueLyser II device and centrifuged at 14,000 rpm for 0.5 min (longer if required). Clear supernatants were then transferred to a new tube on dry ice, samples were then stored at −80 °C until analysis. Analysis of mIns and NAA was performed with Gas Chromatography Mass Spectrometry (GCMS) and for Creatine and Choline using Liquid Chromatography Mass Spectrometry (LCMS). All metabolite values for an individual fetal sample were measured from a single tissue sample to eliminate inter-assay variability. All analysis was run by the Mass Spectrometry Core Facility, Kings College London (KCL, UK).

#### 2.6.3 Gas chromatography mass spectrometry (GCMS) for myo-inositol and NAA

Samples (10ul; Blank, Calibration standard or test samples) were added to tubes containing internal standard-200 (20 μl) + solution (50 μl) (mixture of internal standards (200 μg/ml mIns internal standard, 50 μg/ml NAA internal standard solution). These were evaporated to nearly dry under N2 at 40 ± 5 °C. Pyridine (10 μl) and BSTFA (40 μl) were then added and the samples were vortexed, heated at 80 ± 5 °C for 30 min, cooled down to room temperature and transferred to injection vials. Calibration ranges were 50–2000 μg/ml for mIns(Cat# M01914, Fluorochem Limited, UK; Internal Standard MIns-D6 Cat# 1665997, Toronto Research Chemicals, Canada) and 1–100 μg/ml for NAA (Cat# 00920, SLS, UK). Samples were analysed on an Agilent GCMSD system. GC–MS parameters are detailed in Table 2.

**Table 2:** GCMS and LCMS parameters.

Gas Chromatography (GC)-Mass Spectrometry (MS) Parameters (Myo-Inositol and NAA)		Liquid Chromatography (LC)- Mass Spectrometry (MS) Parameters (Choline and Creatine)	
GAS chromatography (GC)		Liquid chromatography (LC)	
GC Column Injection Temp Constant Gas Flow Injection Mode Split Ratio Injection Volume Run Time	J&W DB-5MSCapillary 30 m × 250 μm × 0.25 μm 280 °C 1 ml/min Split 10:1 1 μL 13.3 min	LC Column LC Column Temp Injection Tray Temp Injection Volume Flow Rate Run Time	Agilent Zorbax Eclipse C18 Rapid Resolution HD 100 × 2.1 mm 1.8 μ 40 °C 10 °C 5 μL 200 μL 6 min
GC oven Temp Program temperature 100 °C/min and hold time 1.0 min, oven ramp: 30 °C/min, temperature 320 °C/min, hold time; 5 min, run time; 13.3 min			
Retention Times *Myo-Inositol* *N-Acetylaspartic Acid* *Myo-Inositol-D6* *Malic Acid-D3*	7.2 min 5.7 min 7.2 min 4.6 min	Retention Times *Choline* *Creatine* *Acetylcholine-D4*	1.2 min 1.3 min 1.3 min


Mass spectometry (MS)		Mass spectometry (MS)	
Source Temperature Quad Temperature Acquisition time	230 °C 150 °C 5–13.3 min	Ionisation Polarity Spray Voltage Capillary Temp Auxiliary Gas Flow Sheath Gas Flow Acquisition time Scan type: Scan Width: Scan Time Data type Numbers of Microscans Collision Gas Pressure: Divert Valve:	Electrospray (ESI) Positive 3500 V 350 °C 0 Arb 50 Arb 6 min SRM 0.200 *m*/*z* 0.150 s Profile 1 1.5 mTorr into Waste: 0–0.3 min, into MS: 3–6 min

#### 2.6.4 Liquid chromatography mass spectrometry (LCMS) for creatine and choline

Samples (100 μl; Blank, Calibration or test samples) were added to individual injection vials containing Internal Standard-2u (50 μl; 2 μg/ml Acetylcholine-D4, Cat#D-1555, CDN Isotopes, Canada) and DS1 (900 μl, contains 0.1% Formic Acid in 50% HPLC grade Acetonitrile), vortexed, and injected into the LCMS system. DS1 was run between high and low concentration samples and between sets of the test sample. Calibration ranges were 10–200 μg/ml for Creatine (Cat# BS-9561E, BioServ UK Limited) and 0.10–35.0 μg/ml for Choline (Cat# 67-48-1, Sigma Aldrich, UK). Samples were analysed on a Thermo Accela Pump and CTC Auto sampler coupled to a Thermo TSQ Quantum Access. LC Solvent A (0.1% Formic Acid in water); LC Solvent B (0.1% Formic Acid in Acetonitrile). LCMS parameters are detailed in Table 2.

### 2.7 Statistical analysis

Statistical analysis was performed using MATLAB (Release: 2017a, The MathWorks, Inc., Natick, MA, USA), and visualised in python 3.7 (www.python.org). Linear mixed-effects models (LME) were used to compare results between the two groups (DS and controls) in both the mass spectrometry and MRS studies, controlling for the covariate PMA at scan as a fixed effect. Cohen's *d* values were calculated to quantify effect sizes and the magnitude of interactions (Cohen, 1988; Rosenthal and Rosnow, 2008). Effect sizes were interpreted as small (Cohen's *d* absolute value ≤ 0.4), medium (0.5–0.7) and large (≥0.80), the sign is indicative of direction of effect (Cohen, 1988). Linear regression and Pearson's correlation tests were also conducted to assess change with PMA at scan separately for each group and main metabolite ratio (MRS) or metabolite concentration (Mass Spectrometry).

## 3 Results

### 3.1 Neonatal *in vivo* MRS

Spectra from 12 neonates with DS and 26 healthy control neonates were analysed; 4 healthy control spectra had low SNR (<8) and the fit failed. In one neonate with DS two spectra were acquired 7 min apart. Since the estimated metabolite concentrations differences for these 2 spectra were relatively small (5% for Cho and NAA, 1% for Cr and Gly and 20% for mIns), the data was averaged.

The characteristics of all term babies are presented in Table 3. Data from all spectra from DS neonates and 22 spectra from healthy neonates are reported in detail below.

**Table 3:** Summary of the DS and Control neonates with interpretable spectra. Congenital Heart Disease (CHD), Gestational age (GA) and post-menstrual age (PMA).

	Down Syndrome neonates	Control neonates
Term babies (n)	10	22
GA at birth, median (range)	36.86 (31.71–38.86) weeks	39.57 (36.57–41.71) weeks
PMA at scan, median (range)	39.15, (37.57–45.57) weeks	42.86, (39.57–43.86) weeks
Males/Females (n)	6/4	14/8
Congenital Heart Disease (n)	7	0

Due to low number of subjects, differences in metabolite concentration ratios between males and females or presence/absence of congenital heart defects (CHD; *n* = 7 in the DS group) were not analysable.

Spectral fits were overall good, with an average ± standard deviation of the signal to noise ratio (SNR) of 17 ± 3 (range 13–22) for controls and 19 ± 3, (range 16–25) for DS neonates. FWHM was 5 ± 1 Hz, (range 4–8 Hz) for controls and 6 ± 1 Hz (range 3–8 Hz) for DS neonates. The % errors on the estimated metabolite concentrations (%SD) were all below 22% for Cho, Cr, NAA (respectively 5 ± 2%, 4 ± 2%, 8 ± 4%) and 16 ± 9% for mIns (all spectra had %SD for Cho, Cr, NAA and mIns <26% except 2 healthy control spectra with %SD > 30%); for Gly the %SDs were 22 ± 12% (<32% for all spectra, except for 4 healthy control spectra > 30%).

**Fig. 2:**
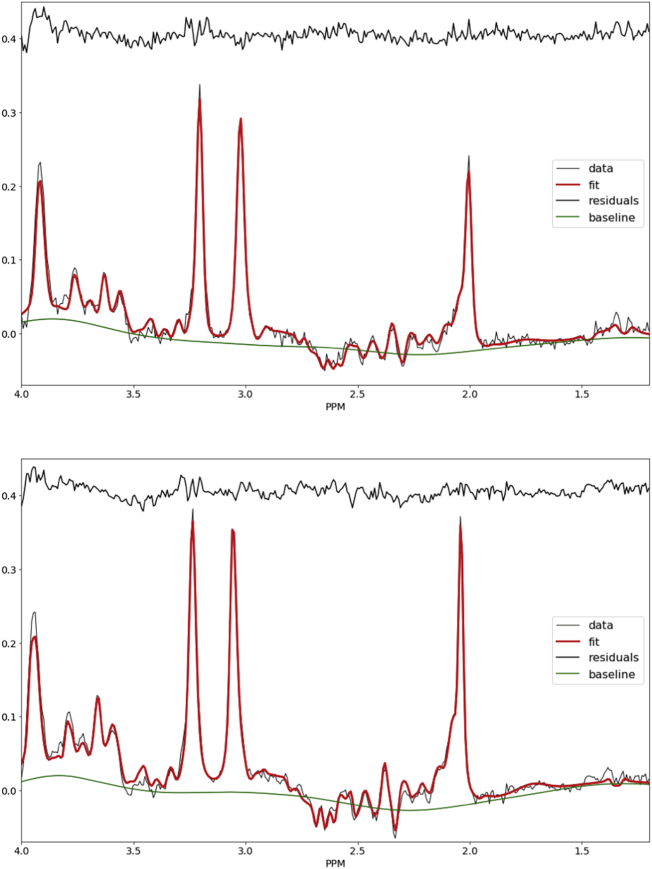
Examples spectra MR spectrum from a control neonate (PMA 42.9 weeks PMA, top) and neonate with DS (number 3 in Table 1, PMA 43.6 weeks, bottom).

Fig. 2A and Fig. 2B show example spectra and fitting for one control and one neonate with DS whose mIns metabolite ratios were close to their respective group mean.

**Fig. 3:**
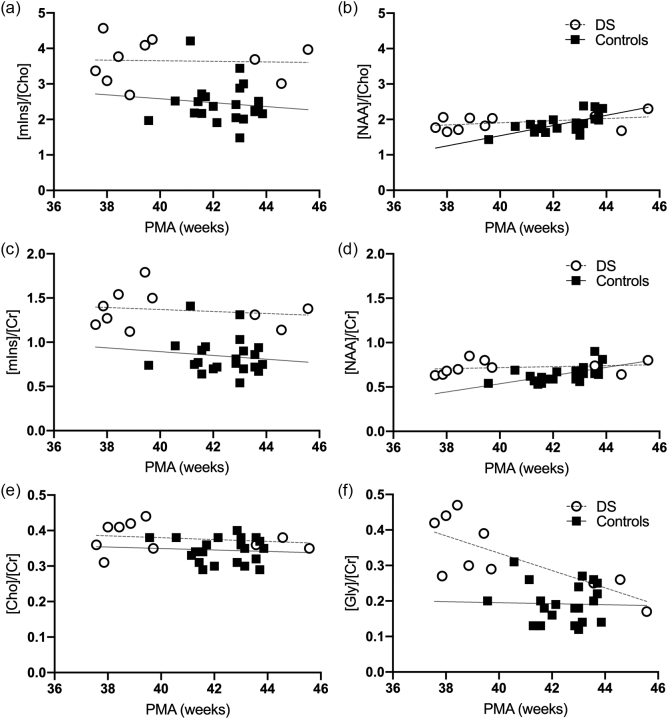
Metabolite ratios in neonates with DS (open circles) and age-matched normal controls (black squares). (a) [mIns]/[Cho], (b) [NAA]/[Cho], (c) [mIns]/[Cr], (d) [NAA]/[Cr], (e) [Cho]/[Cr] (TE 55 ms), and (f) [Gly]/[Cr]. Grey lines indicate linear trends. PMA: Post-menstrual age.

The main metabolite ratios are shown in Fig. 3 for all term neonates. Significant linear correlations with PMA were only found for [NAA]/[Cho] and [NAA]/[Cr] in the control group (*p* < 0.004) and for [Gly]/[Cr] in DS (*p* = 0.012). The linear regression equations and Pearson's correlation coefficients for all main metabolite ratios *vs* PMA are detailed in Supplementary Table 2.

Results of the statistical comparisons between groups, corrected for PMA at scan are shown in Table 4. [mIns]/[Cho] and [mIns]/[Cr] were elevated in DS neonates compared to controls (both *p* < 0.0001, Fig. 3A, C). [Cho]/[Cr] and [Gly]/[Cr] were also significantly increased in DS (*p* < 0.004 and *p* < 0.0005, Fig. 3E, F). There was no significant difference in [NAA]/[Cho] or [NAA]/[Cr] ratios in the DS cohort compared with controls (Fig. 3B, D).

**Table 4:** MRS statistical summary: - LME results, Cohen's *d* values and effect sizes for the DS compared with control cohort. Effect sizes were interpreted as small (Cohen's *d* absolute value ≤ 0.4), medium (0.5–0.7) and large (≥0.8) (Cohen, 1988). *N*-acetylaspartate (NAA), Creatine (Cr), Choline (Cho), Myo-inositol (mIns), Glycine (Gly).

Metabolite concentration ratios	Estimate	Standard error	df	t-Value	p-Value	Effect Size
						*Cohen's d*
[mIns]/[Cho]	−1.16	0.24	29	−4.84	0.00004⁎	−1.80
[mIns]/[Cr]	−0.50	0.08	29	−5.87	0.000002⁎	−2.18
[NAA]/[Cho]	−0.03	0.01	29	−1.92	0.065	−0.71
[NAA]/[Cr]	−0.15	0.09	29	−1.68	0.1	−0.62
[Cho]/[Cr]	−0.11	0.03	29	−3.17	0.0036⁎	−1.18
[Gly]/[Cr]	−0.10	0.03	29	−3.86	0.0005⁎	−1.43

⁎ Significant result, *p* < 0.05.

Spectra from the two preterm DS neonates were not included in the analysis as they were scanned before term equivalent age. DS preterm neonates, number 11 and number 12 (birth GA: 32 and 31.71 weeks; PMA at scan: 32.43 and 34.14) had estimated metabolite concentration ratios as follows: [mIns]/[Cho]: 5.45 and 3.34; [mIns]/[Cr]: 2.89 and 1.92; [NAA]/[Cho]: 1.21 and 1.33; [NAA]/[Cr]: 0.64 and 0.76, [Cho]/[Cr]: 0.53 and 0.57; [Gly]/[Cr]: 0.69 and 0.61 respectively.

### 3.2 Fetal *ex vivo* mass spectrometry

**Fig. 4:**
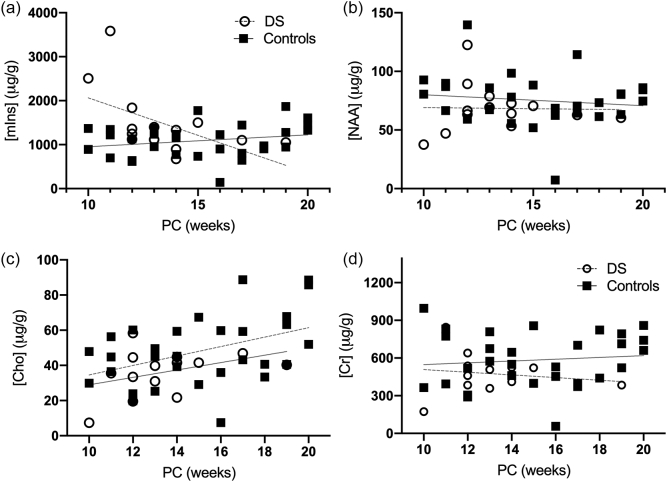
Absolute values for Creatine, Choline, Myo-inositol and NAA in fetal brain tissue for DS (open circles) and age-matched control (black squares) across gestation. PC: Post-conception age. Grey lines indicate linear trends.

We measured the total amount of the metabolites Creatine, Choline, mIns and NAA (Fig. 4; Table 5) in cortical tissue from the fetal brain. 14 fetuses with DS (median (range) - 13.50 (10–19) PCW and 30 control fetuses (15.00 PCW, (10−20) PCW) were used in this study. We found a statistically significant elevation in total mIns in DS fetal brains compared to controls (*p* < 0.05, d = −0.7), when controlled for age (Fig. 4A). This difference appears to be greater in the 10–12 PCW samples analysed. No group differences were observed in Creatine, Choline and NAA. However, the trajectories over gestation appear to be lower in DS as compared to controls. Specifically, mIns and Creatine appears to be on downward trajectories in DS, as compared to the control population. For comparison with the MRS study, ratios were calculated for the mass spectrometry cohort, from the absolute values (Supplementary Table 3). The ratios for [mIns]/[Cho] and [mIns]/[Cr] were found to be significantly different between the two groups (p < 0.05), supporting our MRS findings. However, a statistically significant difference was not detected for the other ratios.

**Table 5:** Mass Spectrometry statistical summary: - LME results, Cohen's *d* values and effect sizes for the DS compared with control cohort. Effect sizes were interpreted as small (Cohen's *d* absolute value ≤ 0.4), medium (0.5–0.7) and large (≥0.8) (Cohen, 1988). *N*-acetylaspartate (NAA), Creatine, Choline and Myo-inositol (mIns).

Metabolites	Estimate	Standard error	df	t value	*p* value	Cohen's d
mIns	−370.8	170.37	41	−2.18	0.04⁎	−0.7
NAA	8.15	7.03	41	1.16	0.25	0.4
Choline	7.81	5.31	41	1.47	0.15	0.5
Creatine	106.07	64.83	41	1.64	0.11	0.5

⁎ Significant result, *p* < 0.05.

## 4 Discussion

In this study, we investigated metabolic profiles in the developing brain with DS, with focus on mIns. To the best of our knowledge, this is the first study to report elevations in both absolute total mIns values *ex vivo* in the human fetal brain with DS and *in vivo* MRS brain data in neonates with DS. We found significant alterations in the metabolite ratios of mIns, [Cho]/[Cr], and [Gly]/[Cr] in the brain of neonates with DS compared to typically developing neonates. We also found that absolute mIns was elevated in fetal brain tissue in DS, prior to 20 weeks when compared to controls, markedly so earlier in gestation at 10 weeks.

Our study found mIns ratios were significantly higher in the DS neonates, compared to the control population in the deep gray matter (as measured in the left basal ganglia and thalamic region). In agreement with an elevation of mIns in DS, our analysis of fetal brain tissue with mass spectrometry also demonstrated elevated mIns levels in the cortex, when compared to controls prior to 20-week gestation, particularly in the earliest ages examined, 10–12 PCW. To date increased absolute mIns levels have not been reported in the human developing fetal DS brain using mass spectrometry. As the early imbalances in mIns levels, from 10 to 12 PCW, may account for the overall observed increase in DS cases, a larger sample size would be needed to determine if there is a consistent, comparative elevation in absolute mIns concentration in DS fetuses.

Myo-inositol is specifically relevant in the pathology of DS, as the NA+/mIns co-transporter (SLC5A3) is located on HSA21 (Berry et al., 1999). Inositol transporters such as sodium-mIns cotransporter-1 (SMIT1), which are encoded by the SLC5A3 gene, are responsible for regulating brain mIns levels, by co-transporting two sodium ions along the concentration gradient to aid active transport of mIns (Fenili et al., 2011; Dai et al., 2016). MIns has been shown to be increased in trisomic cortical neurons (40% compared to diploid), generated from a trisomic mouse model, obtained by breeding normal females to males with heterozygous Robertsonian translocations of chromosome 16 (which shows large synteny with HSA21)(Acevedo et al., 1997). Elevated levels of mIns have also been found in amniotic fluid of human DS pregnancies (15–18 weeks GA) (Santamaria et al., 2014) SMIT1 is overexpressed in human DS and associated with increased intracellular levels of mIns in adults (Shonk and Ross, 1995; Cárdenas et al., 2017). Given the association of mIns transporters and HSA21, our results, likely reflect an elevation in incorporated intracellular mIns concentration.

Myo-Inositol is a glial cell marker and increased levels have been associated with neuroinflammation (Chang et al., 2013). It is speculated that abnormal mIns metabolism may be associated with the initial cognitive impairment in DS as well as being a predisposing factor to AD. Increased concentration of mIns in AD and mild cognitive impairment (without AD) have been supported by histopathologic findings of demyelination and increased gliosis, which is thought to be related to a combination of disintegration of white matter fibres, microglial activation or astrogliosis (Siger et al., 2009). There is increasing evidence from histological studies from mouse models of AD, supporting a link between elevated concentrations of mIns and astrocytic activation (evidenced by an increase in glial fibrillary acidic protein (GFAP) staining), in response to inflammation (Harris et al., 2015).

Increased [mIns]/[Cr] have been identified using MRS, in both gray and white matter, in DS patients (Shonk and Ross, 1995) and non-DS patients (aged 16-to 82), with AD and dementia, and found to precede associated decreases in NAA, atrophy, neuronal loss and cognitive impairment (Öz et al., 2014). Additionally, higher hippocampal concentrations of mIns have been reported in DS adults without dementia, compared to healthy controls (Beacher et al., 2005). Dietary intake has been shown to directly influence plasma levels of mIns, but not those in the CSF, which is thought to be a consequence of global dysregulation in metabolism or of increased dosage of a single gene on HSA21 (Holub, 1986; Shetty et al., 1995). Shetty et al. (1995) placed DS (aged 22–63 years) and control (aged 23–69 years) participants on a low monoaminergic diet for 72 h, prior to collecting samples from a lumbar puncture. They reported a 30–50% higher mIns concentrations in the CSF of DS subjects compared to controls (Shetty et al., 1995; Fisher et al., 2002). As highlighted, the majority of mIns studies have investigated levels in the adult brain, as such, our findings of elevated concentrations in the fetal brain are particularly interesting for further research and as a target for potential interventions.

The association of elevated mIns levels with cognitive impairment in the adult brain, led to a recent clinical trial which aimed to reduce elevated brain mIns levels in adults with DS with scyllo-inositol but found no apparent behavioural or cognitive improvements (Rafii et al., 2018). The authors attribute the result to small participant numbers, short trial duration and the potential practice effects on exploratory cognitive testing. Mouse studies of DS using the Ts65Dn model, have shown promising results in successfully reducing levels of mIns with lithium treatment in the adult mouse, rescuing both synaptic plasticity and memory deficits (Huang et al., 1999; Contestabile et al., 2013). Lithium has been presented as potential treatment option in children with mood disorders, as well as those with intellectual disorders (Yuan et al., 2018). Clinical trials of lithium in children with intellectual disabilities reported positive effects on cognition with only mild, reversible side effects (Yuan et al., 2018), highlighting a potential avenue of research and prospective treatment for DS children with elevated mIns.

We didn't observe any difference in neonatal [NAA]/[Cho] or [NAA]/[Cr] in DS compared to controls. This is consistent with our measurements of NAA from fetal brain tissue prior to 20 weeks gestation. Previous studies in typically developing fetal cohorts have shown increase in [NAA]/[Cho] and [NAA]/[Cr] ratios with increasing GA, coupled with a decrease in [Cho]/[Cr] (Kok et al., 2002; Evangelou et al., 2016). NAA is a critical cerebral metabolite. Increasing levels of NAA are reflective of brain maturation and attributable to dendritic and synaptic development and oligodendroglial proliferation and differentiation (Kato et al., 1997; Battin and Rutherford, 2002). [NAA] is thought to be an early marker of neurodegenerative changes, preceding structural changes as visualised by MRI. Previous studies in adults with DS have reported lower NAA ratios than healthy controls (Śmigielska-Kuzia et al., 2010; Lin et al., 2016). Our MRS data shows that whilst [NAA]/[Cho] and [NAA]/[Cr] increase in our control cohort, they are relatively stable in our small DS cohort; if we speculated that this trend was to continue with brain maturation and PMA, then [NAA]/[Cho] and [NAA]/[Cr] for DS would eventually end up lower than for controls, which would be in agreement with the studies referenced above.

Cho plays a role in lipid membrane synthesis and degradation, and in myelination, and is found in both glia and neurons (Kok et al., 2001; Girard et al., 2006). Adults MRS studies in a healthy ageing population found that Cr levels elevated over time, were thought to be a marker of decreased brain energy metabolism and associated with ageing-related mild cognitive impairment, or dementia in more extreme cases (Ferguson et al., 2002). [Cho]/[Cr] has been shown to slowly decrease with PMA in healthy cases (Evangelou et al., 2016). In our data [Cho]/[Cr] display a mild decreasing trend with increasing PMA in both control and DS cohorts. DS values are slightly higher than control values.

Glycine is a neurotransmitter that is present in the brain during early development. The presence of Glycine receptors in the developing brain is thought to contribute to cell migration and neurotransmitter release (Flint et al., 1998; Avila et al., 2013). We are reporting Glycine concentration ratios because its singlet resonance overlaps with some of the resonances from mIns. In the DS brain, it was shown that at approximately 20 weeks PMA glycine was no different to controls in frontal cortex brain samples, however there is limited understanding of the how glycine expression may change over gestation (Whittle et al., 2007). We found [Gly]/[Cr] significantly increased in DS neonate compared to controls (as well as [Gly]/[Cho]). Whilst further studies will need to confirm the relevance of our findings, it is reassuring that the estimated Glycine concentration is elevated alongside the mIns: considering the spectral overlap, it negates the possibility of the mIns concentration estimates increase being compensated by a Glycine decrease or *vice versa*.

## 5 Limitations

Measuring mIns concentrations is generally challenging due to the complex modulation of its resonances with TE and their overlap with resonances from other metabolites such as Gly, Glu, Gln and Tau. Typically, mIns is estimated using the minimum TE available for the chosen MRS sequence on each particular scanner though different optimisation approaches have been proposed (Kim et al., 2005; Hancu, 2009). Though our TE = 55 ms for PRESS is relatively unusual, this was originally dictated by compatibility with historical protocols. However, analysis of the metabolite basis sets used to fit our neonatal spectra confirmed that with the employed TE1 and TE2 a main mIns singlet-like resonance appears at ~3.64 ppm which facilitates mIns quantification independently from the Gly singlet appearing upfield of it at ~3.57 ppm (a characteristic – and resolvable - mIns/Gly ‘doublet’ appears in most of the spectra analysed in this study) as well as from the complex resonance multiplets of Glu, Gln and Tau appearing in the same spectral region.

The lack of reference MRS measurements of the unsuppressed water signal, did not allow us to report individual metabolite levels; however, whilst absolute metabolite trends cannot be shown, metabolite signal ratios can in some cases prove more sensitive or more measurement-time efficient proxies of abnormalities (Cheong et al., 2006). Moreover, presenting our metabolite concentration ratios *vs* multiple reference metabolites (Cho and Cr) partly mitigates the issue and lends confidence in the robustness of our reported results.

There are also several limitations linked to the nature of working with human fetal tissue samples, that we have taken steps to address in our experimental design. Firstly, whilst a wide range of gestational ages were sampled, wherever possible three individual samples were used per age for the controls. Secondly, in both DS and control the cortical tissue used for the mass spectrometry analysis was selected randomly, to avoid bias in the cortical areas analysed. Thirdly, due to the ages of the samples collected, the majority were collected from surgical, not medical, termination procedures, reducing the effect on tissue metabolites. Finally, once analysed by mass spectrometry, all readings were corrected for cortical tissue weight and DS samples were compared to the age-matched controls. At present we were only able to comment on fresh-frozen fetal tissue, as *in vivo* MRS has not yet been reported on DS fetuses. Future work will aim to optimise MRS sequences in the fetus to account for fetal motion and to obtain a metabolic profile *in vivo* during early brain development.

## 6 Conclusions

In conclusion, we found significantly elevated mIns in the DS fetal brain, and elevated ratios of [mIns]/[Cho] and [mIns]/[Cr], in the developing neonatal brain with DS, compared to a typically developing population. DS is a multifactorial disorder with a wide spectrum of phenotypes attributable to a significant degree of individual variability in genotypes (Karmiloff-Smith et al., 2016; Baburamani et al., 2019). The differences in metabolite ratios may reflect ongoing metabolic deviations that may underlie some of the altered brain volumes previously reported (Patkee et al., 2020) in DS and may be correlated with subsequent cognitive delay and potentially with the development of dementia in later life. Future studies will explore possible correlations with childhood neurodevelopmental assessments in this cohort to ascertain whether metabolite levels predict the degree of early neurocognitive impairment in DS.

## Funding

This work was supported by the 10.13039/501100000265Medical Research Council [MR/K006355/1 and MR/LO11530/1]; 10.13039/501100000833Rosetrees Trust [A1563], Sparks and Great Ormond Street Hospital Children's Charity [V5318]. We also gratefully acknowledge financial support from the 10.13039/100010269Wellcome/EPSRC Centre for Medical Engineering [WT 203148/Z/16/Z], the 10.13039/501100000265Medical Research Council [MR/S025065/1] the 10.13039/501100000272National Institute for Health Research (NIHR) Biomedical Research Centre (BRC) based at Guy's and St Thomas' NHS Foundation Trust and King's College London and supported by the NIHR Clinical Research Facility (CRF) at Guy's and St Thomas'. The Brain Imaging in Babies (BIBS) team additionally acknowledge support from EU-AIMS – a European Innovative Medicines Initiative; and infrastructure support from the 10.13039/501100000272National Institute for Health Research (NIHR) Mental Health Biomedical Research Centre (BRC) at South London and Maudsley NHS Foundation Trust and King's College London.

The views expressed are those of the author(s) and not necessarily those of the NHS, the NIHR or the Department of Health.

## Competing interests

The authors declare that they have no competing interests.

## Appendix A Supplementary data

Supplementary tables Image 1
