# Supplementary material for: Neurometabolite mapping highlights elevated myo-inositol profiles within the developing brain in down syndrome
Source: Neurobiol Dis. 2021 Jun;153:105316. doi: 10.1016/j.nbd.2021.105316 (PMC8039898; doi:10.1016/j.nbd.2021.105316)
Supplement: Supplementary file 1 — Supplementary tables [file mmc1.docx]

**Supplementary Table 1: Case details of fetal brain samples with DS and aged-matched controls.** Age at birth, in post-conceptional weeks (PCW) and sex are listed.

| **Subject ID** | **Age (PCW)** | **Sex (Male/ Female)** |
| --- | --- | --- |
| DS 1 | 10 | F |
| DS 2 | 11 | Unknown |
| DS 3 | 12 | M |
| DS 4 | 12 | F |
| DS 5 | 12 | M |
| DS 6 | 12 | M |
| DS 8 | 13 | F |
| DS 9 | 13 | M |
| DS 10 | 14 | M |
| DS 11 | 14 | M |
| DS 12 | 14 | M |
| DS 13 | 15 | F |
| DS 14 | 17 | M |
| DS 15 | 19 | M |
| Control 1 | 10 | F |
| Control 2 | 10 | F |
| Control 3 | 11 | M |
| Control 4 | 11 | M |
| Control 5 | 11 | M |
| Control 6 | 12 | F |
| Control 7 | 12 | M |
| Control 8 | 12 | M |
| Control 9 | 13 | M |
| Control 10 | 13 | M |
| Control 11 | 13 | M |
| Control 12 | 14 | F |
| Control 13 | 14 | F |
| Control 14 | 14 | M |
| Control 15 | 15 | M |
| Control 16 | 15 | F |
| Control 17 | 16 | F |
| Control 18 | 16 | M |
| Control 19 | 16 | M |
| Control 20 | 17 | F |
| Control 21 | 17 | F |
| Control 22 | 17 | M |
| Control 23 | 18 | M |
| Control 24 | 18 | F |
| Control 25 | 19 | F |
| Control 26 | 19 | M |
| Control 27 | 19 | F |
| Control 28 | 20 | M |
| Control 29 | 20 | F |
| Control 30 | 20 | F |

**Supplementary Table 2: Linear regression, Pearson’s Correlation R2 Coefficients and p values for MRS ratios versus PMA at Scan**

| **Control (n=22)** | **[mIns]/[Cho]** | **[mIns]/[Cr]** | **[Cho]/[Cr]** | **[NAA]/[Cho]** | **[NAA]/[Cr]** | **[Gly]/[Cr]** |
| --- | --- | --- | --- | --- | --- | --- |
| **Linear Regression (y=)** | -0.05519 X + 4.791 | -0.02138 X + 1.749 | -0.002073 X + 0.4324 | 0.1439 X - 4.215 | 0.04618 X - 1.311 | -0.001413 X + 0.2517 |
| **Pearsons r^2^** | 0.01249 | 0.01439 | 0.005040 | 0.4296 | 0.3509 | 0.0008855 |
| **p-value** | 0.62 | 0.59 | 0.75 | 0.0009* | 0.0037* | 0.90 |
| **DS (n=10)** | **[mIns]/[Cho]** | **[mIns]/[Cr]** | **[Cho]/[Cr]** | **[NAA]/[Cho]** | **[NAA]/[Cr]** | **[Gly]/[Cr]** |
| **Linear Regression** | -0.008372 X + 3.988 | -0.01130 X + 1.822 | -0.00261 X + 0.4846 | 0.03008 X + 0.7012 | 0.005594 X + 0.4943 | -0.02439 X + 1.310 |
| **Pearsons r^2^** | 0.001774 | 0.02744 | 0.03879 | 0.1741 | 0.04843 | 0.5659 |
| **p-value** | 0.91 | 0.65 | 0.59 | 0.23 | 0.54 | 0.012* |

**Supplementary Table 3: LME results, Cohen’s *d* values and effect sizes for the DS compared control cohort mass spectrometry ratios, as calculated from the absolute values.** Effect sizes were interpreted as small (Cohen's d value ≤ 0.4), medium (0.5–0.7) and large (≥ 0.8) (Cohen, 1988). *: significant result, p < 0.05.

| **Metabolites (ug/g)** | **Estimate** | **Standard Error** | **df** | **t value** | **p value** | **Cohen's d** |
| --- | --- | --- | --- | --- | --- | --- |
| **[mIns]/[Cho]** | -29.49 | 14.73 | 41 | -2.00 | 0.05* | -0.63 |
| **[mIns]/[Cr]** | -1.48 | 0.58 | 41 | -2.58 | 0.01* | -0.81 |
| **[NAA]/[Cho]** | -0.24 | 0.20 | 41 | -1.21 | 0.23 | -0.38 |
| **[NAA]/[Cr]** | -0.01 | 0.02 | 41 | -0.40 | 0.69 | -0.13 |
| **[Cho]/[Cr]** | 0.00 | 0.01 | 41 | 0.15 | 0.88 | 0.05 |
